# Entropy Stable DGSEM Schemes of Gauss Points Based on Subcell Limiting

**DOI:** 10.3390/e25060911

**Published:** 2023-06-08

**Authors:** Yang Liu, Huajun Zhu, Zhen-Guo Yan, Feiran Jia, Xinlong Feng

**Affiliations:** 1 School of Mathematics and Systems Science, Xinjiang University, Urumqi 830017, China; liuyangscholar@126.com (Y.L.);; 2 State Key Laboratory of Aerodynamics, China Aerodynamics Research and Development Center, Mianyang 621000, China; 3 School of Power and Energy, Northwestern Polytechnical University, Xi’an 710000, China

**Keywords:** high-order methods, Gauss solution points, entropy-stable, subcell limiting, shock capturing

## Abstract

The discontinuous Galerkin spectral element method (DGSEM) is a compact and high-order method applicable to complex meshes. However, the aliasing errors in simulating under-resolved vortex flows and non-physical oscillations in simulating shock waves may lead to instability of the DGSEM. In this paper, an entropy-stable DGSEM (ESDGSEM) based on subcell limiting is proposed to improve the non-linear stability of the method. First, we discuss the stability and resolution of the entropy-stable DGSEM based on different solution points. Second, a provably entropy-stable DGSEM based on subcell limiting is established on Legendre–Gauss (LG) solution points. Numerical experiments demonstrate that the ESDGSEM-LG scheme is superior in non-linear stability and resolution, and ESDGSEM-LG with subcell limiting is robust in shock-capturing.

## 1. Introduction

Computational fluid dynamics (CFD) is a subject that solves the governing equations of fluid dynamics by numerical methods, obtains a discrete quantitative description of the flow field, and predicts the laws of fluid movement based on this description. Non-linear conservation laws are an important research direction in CFD, so many scholars have focused on using high-order methods to solve these problems. Relative to low-order methods, high-order methods have the advantages of less dissipation, less dispersion, and higher accuracy, and can describe the laws of fluid motion more accurately [1]. Therefore, high-order methods have become important approaches for scientists and engineers to pursue accurate predictions of flow problems.

The discontinuous Galerkin (DG) method cannot only achieve a high-order of accuracy but also adapt to complex grids and geometry configurations, which is of great significance for the real characterization of flow laws [2,3]. The main idea of this method is to discretize the whole computational domain into a finite number of cells and approximate the solutions on the cells using a polynomial space to represent the solutions [4]. Although great progress has been made in applying high-order DG methods to simulate non-linear problems in recent years, the instability associated with the discrete scheme still constrains the development and application of the high-order DG method. The stability of the high-order DG method is mainly degraded by aliasing errors and shock waves [5,6,7]. There are two main reasons for the aliasing errors: first, using an insufficient Gauss quadrature; and second, the error caused by the approximations between the derivative of the interpolation and the interpolation of the derivative. Those instabilities are improved through various stabilization techniques, including filtering [8], split form [9], over-integration [10], etc. The instability caused by shock waves occurs mainly because a high-order scheme generally adopts high-order distribution approximation, which tends to generate many “non-physically relevant” solutions. In response to this phenomenon, we mainly adopt the following methods: the entropy-stable scheme [11], the hybrid scheme [12], and the limiter [13].

As it is significantly difficult to satisfy entropy dissipation or energy conservation, it is usually impossible to guarantee the physical meaning of the numerical solution. However, high-order methods using the entropy-stable strategy not only have high precision in solving this problem but have also become an effective means to solve hyperbolic conservative laws [14,15]. Osher and Tadmor proposed, for the first time, a entropy conservative and entropy-stable strategy in the finite difference method [16]. The key to this strategy is to construct a dissipation operator of flux that satisfies the entropy conditions. Later, Tadmor et al. constructed an entropy-stable scheme with arbitrary-order accuracy, combining high-order entropy conservative flux with arbitrary-order numerical dissipation operators of polynomial reconstruction [17]. In recent years, entropy-stable schemes have been extended to curved meshes and simple meshes based on different solution points [18,19]. Shu et al. extended the entropy-stable scheme to the discontinuous Galerkin spectral element method (DGSEM) of the Legendre–Gauss–Lobatto (LGL) solution points in unstructured grids and obtained good results [20]. The implementation of these schemes benefits from the approximation of the first derivative of flux at the LGL solution points. Although the high-order entropy-stable scheme established at LGL solution points has better stability and resolution, it still produces a high-order aliasing effect and low resolution. Furthermore, when taking the same degrees of freedom, the high-order entropy-stabilization scheme of Legendre–Gauss (LG) solution points has higher precision and resolution. Therefore, it is necessary to develop a high-order entropy-stabilization scheme based on LG solution points. Recently, Carpenter et al. proposed how to effectively extend the construction of a semi-discrete entropy-stable scheme to LG solution points by combining summation-by-parts (SBP) finite difference operators [10,21,22].

The entropy-stable DGSEM has been greatly developed in recent years and can provide better robustness for the computational simulation of fluid motion than other schemes. However, an additional stabilization mechanism is still needed for the strong shock-wave problem. In recent years, a hybrid method based on different precision or different discrete schemes has been developed. This method ensures the stability and accuracy of the high-order scheme by reducing the polynomial order in the shock region and refining the mesh [23,24]. Recently, this method has been widely used in conjunction with the DG method. The solution region is divided into smooth and troubled regions, in which shock-capturing is carried out. In 2014, Dumbser et al. proposed subcell limiting for DG methods in [25]. In 2016, Dumbser extended the method to general unstructured triangular methods [26]. In 2017, Sonntag and Munz proposed a subcell-limited shock-capture strategy using two schemes, DG and FV, improving the resolution without increasing the number of degrees of freedom (DOFs) [27]. In 2021, Krais et al. combined the DG scheme with the idea of discrete subcell FV to capture shock waves [28]. The above are all high-order methods established based on LGL solution points. This is due to the precision of the high-order scheme of the LG solution points being higher than that of LGL solution points. In 2022, Zhu et al. developed the subcell CNNW limiting for the CPR method with LG solution points in [29]. However, although these shock-capturing schemes can capture discontinuous solutions, they may still produce non-physical solutions for strong shock-wave problems because they do not consider entropy stability. In view of this situation, Hennemann established a shock-capturing scheme of subcells with proven entropy stability at the LGL solution points, which could not only carry out shock wave capturing but also satisfy the entropy conditions over the whole calculation process [30]. Compared with the traditional high-order DG method, the high-order scheme of the entropy-stable strategy can provide better robustness in computational simulation. However, the high-order entropy-stable scheme established at LGL solution points still yields lower resolution and stability for problems such as under-resolved flow problems and less-resolved density flows [31,32,33].

To solve the above problems, we investigated the stability and resolution of high-order entropy-stable schemes based on different solution points, and subcell shock-capturing technology with provable entropy stability based on LG points is developed for hyperbolic conservation laws in this paper. The main work of this paper contains the following:

1. High-order entropy-stable schemes based on different solution points are discussed. Although high-order schemes have been shown to satisfy the discrete entropy condition. They have different stability properties for the same test case. The stability and resolution of the high-order schemes are analysed in the context of the Kelvin–Helmholtz instability problem and under-resolved vortical flows.
2. A provably entropy-stable high-order scheme based on subcell limiting is proposed for hyperbolic conservation laws. First, we use an indicator considering the modal decay of the polynomial representation based on an extended stencil to detect troubled cells. Second, the cells to be solved are divided into troubled and smooth cells [34,35]. To ensure that the hybrid scheme is entropy-stable, we choose the same entropy-stable or entropy conservative numerical flux. The hybrid scheme can be implemented according to the magnitude of the troubled cell indicator to mediate the change from a smooth region to a discontinuous region.

This paper is organized as follows. In Section 2, we briefly review the derivation of continuous entropy inequalities for conservation laws. In Section 3, we describe how to construct high-order entropy-stable DGSEM schemes of conservation laws using different solution points. In Section 4, high-order entropy-stable DGSEM schemes of Gaussian solution points based on subcell limiting are proposed, and Section 5 presents numerical results that confirm the effectiveness of the proposed method for smooth and discontinuous flows based on compressible Euler equations.

## 2. A Brief Review of the Basic Theory

### 2.1. Conservation Laws

Consider the two-dimensional conservation laws in physical space

(1) $$\frac{\partial U}{\partial t}+\frac{\partial F}{\partial x}+\frac{\partial G}{\partial y}=0.$$

where, *U* is the conserved variable vector, and *F* and *G* are the convection fluxes

(2) $$\begin{array}{c} U=\left[\begin{array}{c} \rho \\ \rho u\\ \rho v\\ E \end{array}\right],F=\left[\begin{array}{c} \rho u\\ \rho u^{2}+p\\ \rho uv\\ u(E+p) \end{array}\right],G=\left[\begin{array}{c} \rho v\\ \rho uv\\ \rho v^{2}+p\\ v(E+p) \end{array}\right],\\ E=\frac{p}{\gamma -1}+\frac{1}{2}\rho \left(u^{2}+v^{2}\right). \end{array}$$

where ρ is the density, *u* and *v* are velocities in the directions of *x* and *y*, respectively, *p* is the pressure and *E* is the total internal energy. For an ideal fluid, the specific heat ratio (gamma) is 1.4.

### 2.2. Entropy Inequality

To introduce the concept of entropy inequality, this section starts from the general one-dimensional conservation law

(3) $$\frac{\partial U}{\partial t}+\frac{\partial F}{\partial x}=0,$$

where *U* is a conservative variable and F(U) is the flux vector. The entropy of the solution of the non-linear conservation equation without diffusion only increases near discontinuities. Therefore, the entropy can be used as a criterion to find the physical solution from mathematically possible weak solutions. To this end, we introduce the entropy function S(U) and entropy variable v(U), which satisfy the following relationship [20]

(4) $$v\left(U\right)=\frac{\partial S(U)}{\partial U}.$$

where S(U) is convex, and the entropy flux is defined as $F_{i}^{s}\left(U\right)$. Furthermore, the entropy potential is defined as $\psi_{i}\left(U\right)=v^{T}F_{i}\left(U\right)-F_{i}^{s}\left(U\right)$. The entropy function and entropy flux are defined as an entropy pair. The entropy equation is satisfied in smooth regions as

(5) $$\begin{array}{c} \frac{\partial S(U)}{\partial t}+\sum_{i=1}^{d}\frac{\partial F_{i}^{s}\left(U\right)}{\partial x_{i}}=0. \end{array}$$

while the entropy inequality condition is satisfied in the discontinuous region as

(6) $$\frac{\partial S(U)}{\partial t}+\sum_{i=1}^{d}\frac{\partial F_{i}^{s}\left(U\right)}{\partial x_{i}}\leq 0.$$

## 3. The Hybrid Scheme

In this section, a provably entropy-stable shock-capturing scheme based on the LG solution points is proposed. Firstly, entropy-stable DGSEM based on LG and LGL solution points is briefly introduced. Furthermore, the main method divides the computational cells into troubled and smooth cells. An entropy-stable scheme based on the subcell limiting technique is developed. For the limiting technique, troubled cell indicators are used to detect troubled cells and then a low-order scheme is adopted to solve troubled cells. The troubled cell is divided into several subcells based on the Gaussian quadrature weights $W_{j}$.

### 3.1. ESDGSEM Schemes

The high-order entropy-stable DGSEM based on the LGL solution points is introduced in [20], and the entropy-stable DGSEM scheme based on LG solution points is introduced in [21]. The DGSEM scheme is a high-order DG method in split form, which has better stability than the traditional DG method [10]. Assuming that the computational domain is [a,b] and divided into *C* cells, the *k*th cell is $H^{k}=\left[x^{k},x^{k+1}\right],k=1,\cdots ,K$. The solutions in each cell are represented by an *N*th-order polynomial space with basis functions $\psi_{j}$, which are Lagrange functions on $N_{q}=N+1$ solution points. The solutions are approximated by

(7) $$U^{k}\left(x,t\right)=\sum_{j=1}^{N+1}U^{k}\left(x_{j}^{k},t\right)\psi_{j}\left(x_{j}^{k}\right).$$

The weak and strong forms of the conservation equations can be obtained by selecting the same trial and test functions $\psi_{j}$. Here we focus on the strong form of conservation equations. Within the *k*-th cell, *j*-th LGL solution points are expressed as $x_{j}^{k}$. The cells in physical space are mapped to the standard cell $I_{k}$ of [−1, 1]. $\xi_{j}$ represents the coordinates of the solution points in the standard cell and the corresponding quadrature weights are ${\left\{w_{j}\right\}}_{j=1}^{N+1}$. We choose the Lagrangian function as the interpolation basis function. The basis function is of the following form

(8) $$l_{j}\left(\xi \right)=\prod_{\begin{array}{c} l=1\\ l\neq j \end{array}}^{N_{q}}\frac{\xi -\xi_{l}}{\xi_{j}-\xi_{l}},\;\delta_{ij}=\left\{\begin{array}{cc} 1 & i=j\\ 0 & i\neq j \end{array},\right.$$

The continuous integration is approximated by the discrete inner product ${\langle u,v\rangle }_{\omega }$ as

(9) $$\begin{array}{c} M_{ij}=\int_{-1}^{1}l_{i}\left(\xi \right)l_{j}\left(\xi \right)d\xi \approx \sum_{k=1}^{N_{q}}l_{i}\left(\xi_{k}\right)l_{j}\left(\xi_{k}\right)w_{i}=\delta_{ij}w_{i},\\ Q_{ij}=\int_{-1}^{1}l_{i}\left(\xi \right)l_{j}^{\prime }\left(\xi \right)d\xi . \end{array}$$

To introduce DGSEM based on LGL solution points, we briefly recall the generalized SBP strategy.

**Theorem** **1.** *Q=MD satisfies the generalized SBP property*

(10) $$Q=V_{f}^{T}BV_{f}-Q^{T},\;B=\left[\begin{array}{cc} -1 & \\ \; & 1 \end{array}\right],$$

*where D is the differential matrix, M is the weight, and $V_{f}$ is the boundary interpolation matrix, which can be used to extract the solution point values related to the left and right endpoints [21].*

Theorem 1 can be applied to approximate first-order derivatives for both LGL and LG solution points. Its main function is to simulate the properties of SBP for first-order derivatives, and when combined with the DG method, the high-order scheme can provide higher accuracy and better stability. More importantly, the generalized SBP operator based on the quadrature rule can be applied to non-equidistant LGL and LG solution points, making the accuracy at the boundary and internal compatible, and provide a useful tool for LGL and LG solution points to establish a high-order DG scheme.

We define the left and right state variables at the boundary as $U_{L}^{k}=U^{k-1}\left(\xi_{R}\right),U_{R}^{k}=U^{k}\left(\xi_{L}\right)$. Furthermore, by discretizing the continuous and conservative integral terms with the generalized SBP operators, the high-order DGSEM scheme based on LGL solution points (DGSEM-LGL) [11] can be obtained

(11) $$JM\frac{dU}{dt}+V_{h}^{T}\left(\left(Q-Q^{T}\right)\circ F\right)1+V_{f}^{T}Bf^{*}=0.$$

where *J* is the Jacobian matrix of the mapping of local cells. $V_{h}$ contains the interpolation matrix of the interior and boundary of the form $\left(I,V_{f}\right)$. *B* is the boundary matrix, and $M=\mathrm{diag}\left\{w_{0},\cdots ,w_{K}\right\}$ is the weight matrix composed of diagonal Gaussian quadrature weights. Here, “∘” represents the Hadamard product, and $f^{*}$ is the interface numerical flux matrix containing the left and right end of the cells

(12) $$f^{*}={\left(f_{L}^{*},f_{R}^{*}\right)}^{T},\;f_{L}^{*}=\left(U_{L}^{k},U_{R}^{k}\right),\;f_{R}^{*}=\left(U_{L}^{k+1},U_{R}^{k+1}\right).$$

Equation (11) is the rewritten DGSEM scheme using the generalized SBP operators, which is conducive to comparison with the entropy-stable discrete scheme. We observe that the DGSEM scheme can satisfy the energy-conservation or entropy-stability condition if the conserved flux differential is rewritten appropriately. To introduce the high-order entropy-stable scheme, two important definitions of entropy-conservative and entropy-stable numerical flux are given below.

**Definition** **1.** *A consistent, symmetric two-point numerical flux $f_{s}\left(u_{L},u_{R}\right)$ is entropy-conservative for a given entropy function [21] if*

(13) $$\begin{array}{cc} \; & f_{S}\left(u,u\right)=f\left(u\right),\\ \; & f_{S}\left(u_{L},u_{R}\right)=f_{S}\left(u_{R},u_{L}\right),\\ \; & {\left(v_{R}-v_{L}\right)}^{T}f_{S}\left(u_{L},u_{R}\right)=\psi_{R}-\psi_{L}. \end{array}$$

*where $\left\{v_{L},v_{R}\right\}$ and $\left\{\psi_{L},\psi_{R}\right\}$ are entropy variables and entropy potential at the left and right states, respectively.*

**Definition** **2.** *A consistent, symmetric two-point numerical flux $f_{s}\left(u_{L},u_{R}\right)$ is entropy-stable for a given entropy function [21] if*

(14) $${\left(v_{R}-v_{L}\right)}^{T}f_{S}\left(u_{L},u_{R}\right)-\left(\psi_{R}-\psi_{L}\right)\leq 0.$$

Equation (13) shows that the entropy-conservative flux is always in dynamic equilibrium with the entropy variables and entropy potential, which causes the whole flow field to maintain strict entropy conservation. In Equation (14), the entropy-stable flux is not strictly controlled such that entropy must be in a dynamic conservation, but the entropy can be in a non-increasing state in the whole flow field.

The construction of high-order entropy-stable schemes based on high-order DGSEM schemes relies on entropy conservative and entropy-stable fluxes and generalized by SBP operators that can accurately approximate the first derivative of any function. The most important aspect is that the high-order flux derivatives can be discretized approximately by entropy flux and generalized by SBP operators through the flux difference. Since the cells in the high-order DG method are not continuous with each other, the total entropy is always in a state of entropy increase at the interface. To suppress the non-physical oscillation, an appropriate entropy dissipation term should be added to the entropy conservative scheme to ensure that the high-order scheme is entropy-stable. Therefore, considering the cell boundary conditions, entropy-stable discrete schemes based on LGL and LG solution points are established.

The high-order entropy-stable scheme based on LGL solution points (ESDGSEM-LGL) [11] is expressed as

(15) $$\begin{array}{cc} \; & JM\frac{dU}{dt}+V_{h}^{T}\left(\left(Q-Q^{T}\right)\circ F_{s}\right)1+V_{f}^{T}Bf^{*}=0,\\ \; & {\left(F_{s}\right)}_{ij}=f_{s}\left(U_{i},U_{j}\right),\;1\leq i,j\leq N_{q}, \end{array}$$

where ${\left(F_{s}\right)}_{ij}$ is the numerical flux of the two-point entropy conservative displayed in Appendix A. The solution obtained by the ESDGSEM-LGL scheme has a physical meaning, in contrast to the DGSEM-LGL scheme.

The precision of the high-order scheme based on the LG solution points is also higher. Therefore, it is necessary to establish the high-order entropy-stable scheme on the LG solution points [11]. Instead of approximating the variables at the boundary directly by interpolation of the original variables, establishing the high-order entropy-stable scheme at the LG solution points requires converting the original variables into entropy variables and using boundary interpolation and L2 projection to re-estimate the variables at the boundary.

(16) $$\tilde{v}=v\left(U\right),\;\tilde{U}=U\left(V_{h}\tilde{v}\right).$$

This process is defined as “entropy projected” [11]. Here, $\tilde{U}$ contains the conservative variable values on the cell interface and inside, and $\tilde{v}$ are the entropy variables values at the solution points.

The high-order entropy-stable discrete scheme for LG solution points (ESDGSEM-LG) [11] is expressed as

(17) $$\begin{array}{cc} \; & JM\frac{dU}{dt}+V_{h}^{T}\left(\left(Q_{h}-Q_{h}^{T}\right)\circ F_{s}\right)1+V_{f}^{T}Bf^{*}=0,\\ \; & {\left(F_{s}\right)}_{ij}=f_{s}\left({\tilde{U}}_{i},{\tilde{U}}_{j}\right),\;1\leq i,j\leq N_{q}. \end{array}$$

which is different from the high-order entropy-stable scheme established at the LGL solution points. Here, $l_{j}\left(\xi \right)$ and $V_{f}$ are the Lagrange basis function and boundary interpolation matrix of the LG solution points, respectively. $Q_{h}$ satisfies the SBP property and can be expressed as

(18) $$V_{f}=\left[\begin{array}{ccc} l_{1}(-1, & \cdots & ,l_{N_{q}}\left(-1\right)\\ l_{1}\left(1\right), & \cdots & ,l_{N_{q}}\left(1\right) \end{array}\right],$$

(19) $$Q_{h}=\frac{1}{2}\left[\begin{array}{cc} 2Q-V_{f}^{T}BV_{f} & V_{f}^{T}B\\ -BV_{f} & B \end{array}\right].$$

The generalized SBP operators can not only effect on the internal integration term but also the boundary integration term. The flux at the internal solution points can be obtained directly by approximation, but the approximation of the flux at the boundary must combine the interpolation and boundary correction matrices. For the above two methods of point selection, if the numerical flux is an entropy-stable flux, the entropy-stable discrete scheme is obtained. The Lax–Friedrichs flux (LLF), HLL flux, and penalty matrix can increase the entropy dissipation of the discrete scheme and meet the entropy-stable flux condition.

### 3.2. Entropy-Stable Scheme Based on the Subcell Limiting Technique

In this subsection, the ESDGSEM based on LG solution points is combined with the subcell limiting technique [34,36]. Firstly, the highest modal decay indicator based on the “extended” template (MDHE indicator) proposed by Zhu [37,38] is applied to detect troubled cells.

(20) $$E^{*}=\max\left(\frac{m_{N^{*}}^{2}}{\sum_{j=1}^{N^{*}}m_{j}^{2}},\frac{m_{N^{*}-1}^{2}}{\sum_{j=1}^{N^{*}-1}m_{j}^{2}}\right),$$

where ${\left\{m_{j}\right\}}_{j=1}^{N^{*}}$ is the modal coefficient. The threshold value

(21) $$T^{*}\left(N\right)=a\cdot 10^{-1.8{\left(N^{*}+1\right)}^{\frac{1}{4}}},$$

is used to determine whether the cell is a troubled cell by Equations (20) and (21). Here, different *a* values are used in the numerical tests. If $E^{*}\geq T^{*}$, the cell is a troubled cell; otherwise, it is a smooth cell.

ESDGSEM based on subcell limiting is in fact a hybrid scheme. Discrete conservation laws and entropy stability will be proven for the hybrid scheme in Section 3.2.1 and Section 3.2.2, respectively. Before the proof, we shows the connection between the ESDGSEM and subcell low-order schemes.

We give the discrete form of the low-order of the subcell at the LG solution points as

(22) $$J\frac{\partial U_{j}^{SL}}{\partial t}M_{j}=-\left(f_{j,j+1}^{*}-f_{j-1,j}^{*}\right).$$

Each subcell has a single finite volume. Constant distribution is considered the solution point values $U_{j}$ and j∈{0,⋯,N} can be explained as averages of the subcells. Here, $f_{j,j+1}^{*}=f^{*}\left(U_{j},U_{j+1}\right)$ is the interface flux of the two neighbouring cells.

We give a high-order scheme at LG solution points as

(23) $$\begin{array}{cc} \; & JM\frac{dU^{DG}}{dt}+V_{h}^{T}\left(\left(Q_{h}-Q_{h}^{T}\right)\circ F_{s}\right)1+V_{f}^{T}Bf^{*}=0,\\ \; & {\left(F_{s}\right)}_{ij}=f_{s}\left({\tilde{U}}_{i},{\tilde{U}}_{j}\right),\;1\leq i,j\leq N_{q}. \end{array}$$

The hybrid scheme switches to a low-order scheme when there is a troubled cell and to a high-order scheme if the cell is a smoothed cell. To more intuitively describe how the hybrid scheme works, we combine the high- and low-order discrete schemes together as

(24) $$R:=a^{*}\sum_{j=0}^{N_{q}}R_{j}^{SL}+\left(1-a^{*}\right)R^{DG}.$$

where *R* is the residual in the discrete scheme, and $a^{*}$ is the switching coefficient, which can take a value of only 0 or 1. The selection of $a^{*}$ depends on the shock detector. If troubled cells are detected, $a^{*}=1$; otherwise, $a^{*}=0$. Equation (24) can be expanded as

(25) $$J\frac{\partial U}{\partial t}M=a^{*}\left(-\left(f_{N,R}^{*}-f_{L,0}^{*}\right)\right)-\left(1-a^{*}\right)\left(V_{h}^{T}\left(\left(Q_{h}-Q_{h}^{T}\right)\circ F_{s}\right)+V_{f}^{T}Bf^{*}\right).$$

By rewriting this hybrid scheme, we can realize the connection between a high-order scheme and a low-order scheme. If the neighbouring cells are all smooth cells or troubled cells, there is no scheme switching, and each single scheme satisfies discrete conservation laws. However, if the neighbouring cells of smooth cells are troubled cells, the conservation laws and entropy stability of the hybrid scheme need to be carefully discussed. We consider only the case where the troubled cells and smooth cells are adjacent.

#### 3.2.1. Conservation of the Hybrid Scheme

Assume that the left side is a smooth cell defined in the $D_{1}$ domain, and the right side is a troubled cell defined in the $D_{2}$ domain.

The high-order scheme for the $D_{1}$ domain is as follow

(26) $$\begin{array}{cc} JM\frac{\partial U^{DG}}{\partial t} & =-\left(V_{h}^{T}\left(\left(Q_{h}-Q_{h}^{T}\right)\circ F_{s}\right)1+V_{f}^{T}Bf^{*}\right)\\ \; & =-\left(V_{h}^{T}\left(2\left(S_{h}+\frac{1}{2}B_{h}-B_{h}\right)\circ F_{s}\right)1+V_{f}^{T}Bf^{*}\right)\\ \; & =-2V_{h}^{T}\left(S_{h}\circ F_{s}\right)1+V_{h}^{T}\left(B_{h}\circ F_{s}\right)-V_{f}^{T}Bf^{*}\\ \; & =-V_{h}^{T}2\left(S_{h}\circ F_{s}\right)1+V_{h}^{T}\left(B_{h}\circ F_{s}\right)-V_{f}^{T}Bf^{*}. \end{array}$$

where $Q_{h}$ satisfies the SBP properties $Q_{h}+Q_{h}^{T}=B_{h}$ and $Q_{h}=S_{h}+\frac{1}{2}B_{h}$. Then, it can be easily proven that the discrete conservation law is

(27) $$\begin{array}{cc} \frac{\partial \left(\int_{}U^{DG}\right)}{\partial t} & =J\frac{\partial \left(\int_{-1}^{1}Ud\xi \right)}{\partial t}=J\frac{\partial \left(\sum_{j=0}^{K}U_{j}W_{j}\right)}{\partial t}=J\left(\sum_{j=0}^{K}W_{j}\frac{\partial U^{DG}}{\partial t}\right)\\ \; & =-\sum_{j=0}^{K}\sum_{i=0}^{K+3}2{\left(V_{h}^{T}\right)}_{ji}{\left(S_{h}\right)}_{ji}{\left(F_{s}\right)}_{ji}+\sum_{j=0}^{K}\sum_{i=0}^{K+3}{\left(V_{h}^{T}\right)}_{ji}{\left(B_{h}\right)}_{ji}{\left(F_{s}\right)}_{ji}\\ \; & -\sum_{j=0}^{K}\sum_{i=0}^{K+3}{\left(V_{h}^{T}\right)}_{ji}{\left(B_{h}\right)}_{ji}{\left(F^{*}\right)}_{i1}\\ \; & =-\sum_{j=0}^{K}\sum_{i=0}^{K+3}{\left(V_{h}^{T}\right)}_{ji}\left({\left(S_{h}\right)}_{ji}+{\left(S_{h}\right)}_{ij}\right){\left(F_{s}\right)}_{ji}\\ \; & +\sum_{j=0}^{K}\sum_{i=0}^{K+3}{\left(V_{h}^{T}\right)}_{ji}\left({\left(B_{h}\right)}_{ji}{\left(F_{s}\right)}_{ji}-{\left(B_{h}\right)}_{ji}{\left(F^{*}\right)}_{i1}\right)\\ \; & =-\sum_{j=0}^{K}\sum_{i=0}^{K+3}{\left(V_{h}^{T}\right)}_{ji}{\left(B_{h}\right)}_{ji}{\left(F_{s}\right)}_{ji}\\ \; & +\sum_{j=0}^{K}\sum_{i=0}^{K+3}{\left(V_{h}^{T}\right)}_{ji}{\left(B_{h}\right)}_{ji}\left({\left(F_{s}\right)}_{ji}-{\left(F^{*}\right)}_{i1}\right)\\ \; & =\sum_{i=0}^{K+3}{\left(V_{h}^{T}\right)}_{ii}{\left(B_{h}\right)}_{ii}{\left(F^{*}\right)}_{i1}\\ \; & =-\left(f_{R}^{*}-f_{L}^{*}\right). \end{array}$$

where $F^{*}={\left(0,0,\cdots ,0,f_{L}^{*},f_{R}^{*}\right)}^{T}$. Equation (27) can be satisfied for each solution point in the interval of $D_{2}$, thus we can obtain

(28) $$\begin{array}{cc} \frac{\partial \left(\int_{}U^{SL}dx\right)}{\partial t} & =J\frac{\partial \left(\int_{-1}^{1}Ud\xi \right)}{\partial t}=J\frac{\partial \left(\sum_{j=0}^{K}U_{j}W_{j}\right)}{\partial t}=J\left(\sum_{j=0}^{K}W_{j}\frac{\partial U_{j}^{SL}}{\partial t}\right)\\ \; & =J\frac{\partial \left(\sum_{j=0}^{K}U_{j}^{SL}W_{j}\right)}{\partial t}=J\left(\sum_{j=0}^{K}W_{j}\frac{\partial U_{j}^{SL}}{\partial t}\right)\\ \; & =J\left(\frac{1}{J}\sum_{j=0}^{K}W_{j}\left(\frac{f_{j,j+1}^{*}-f_{j-1,j}^{*}}{W_{j}}\right)\right)=\sum_{j=0}^{K}-\left(f_{j,j+1}^{*}-f_{j-1,j}^{*}\right)\\ \; & =-\left(f_{N,R}^{*}-f_{L,0}^{*}\right). \end{array}$$

Then, the high- and low-order scheme have the same form of discrete conservation law. Therefore, we sum the discrete conservations of the intervals $D_{1}$ and $D_{2}$ to obtain the discrete conservation of the hybrid scheme as

(29) $$\begin{array}{cc} \frac{\partial \left(\int_{D_{1}}U^{DG}dx+\int_{D_{2}}U^{SL}dx\right)}{\partial t} & =J\frac{\partial \left(\int_{-1}^{1}U^{DG}d\xi +\int_{-1}^{1}U^{SL}d\xi \right)}{\partial t}\\ \; & =-\left(f_{R}^{*}-f_{L}^{*}\right)-\left(f_{N,R}^{*}-f_{L,0}^{*}\right)\\ \; & =-\left(f_{R}^{*}-f_{L}^{*}+f_{N,R}^{*}-f_{L,0}^{*}\right). \end{array}$$

Equation (29) shows that the hybrid scheme satisfies the conservation condition if and only if $f_{L}^{*}=f_{N,R}^{*}$, indicating that when the same numerical flux is selected for the high- and low-order schemes, the hybrid scheme satisfies the conservation laws. Therefore, the same numerical flux will be chosen at the interface in the numerical tests below.

#### 3.2.2. Entropy Stability of the Hybrid Scheme

The conservation proof of the hybrid scheme is mainly given in the previous section, but it cannot guarantee that the scheme can satisfy the entropy condition. We know that the total entropy of a cell is obtained from the sum of the product of the entropy variable and solution points. Therefore, we analyse the evolution of the total entropy of each cell in the shock-capture scheme over time to verify whether the hybrid scheme meets the entropy condition.

For troubled cells, a low-order scheme is adopted. If the low-order scheme meets the entropy condition in the $D_{2}$ interval, then

(30) $$JM\frac{\partial S^{SL}}{\partial t}=-\left(v\left(1\right)f_{N,R}^{*}-v\left(-1\right)f_{L,0}^{*}-\psi_{N,R}+\psi_{L,0}\right).$$

The entropy of each cell is obtained by

(31) $$JM_{j}v_{j}\frac{\partial U_{j}^{SL}}{\partial t}=-v_{j}\left(f_{j,j+1}^{*}-f_{j-1,j}^{*}\right).$$

Then, we can obtain

(32) $$\begin{array}{cc} \sum_{j=0}^{K}JM_{j}\frac{\partial S_{j}^{SL}}{\partial t} & =\sum_{j=0}^{K}-v_{j}\left(f_{j,j+1}^{*}-f_{j-1,j}^{*}\right)\\ \; & =-\left(-v_{0}f_{L,0}^{*}+v_{N}f_{N,R}^{*}-\psi_{N,R}+\psi_{L,0}\right). \end{array}$$

where $v_{0}=v_{L,0},v_{N}=v_{N,R}$. It can be seen from Equation (32) that if the numerical flux of the troubled cells is an entropy-stable numerical flux, then the low-order discrete scheme satisfies the entropy condition.

For smooth cells, we consider the total entropy of each smooth cell, so we multiply both sides of Equation (23) by the entropy variable. We now have

(33) $$\begin{array}{cc} JMv^{T}\frac{dU^{DG}}{dt} & \left.=-\left(v^{T}V_{h}^{T}\left(\left(Q_{h}-Q_{h}^{T}\right)\circ F_{s}\right)1\right)+v^{T}V_{f}^{T}Bf^{*}\right)\\ JM\frac{dS^{DG}}{dt} & \left.=-\left(v^{T}V_{h}^{T}\left(\left(Q_{h}-Q_{h}^{T}\right)\circ F_{s}\right)1\right)+v^{T}V_{f}^{T}Bf^{*}\right)\\ \; & \left.=-\left(2v^{T}V_{h}^{T}\right)\left(\left(S_{h}\circ F_{s}\right)1+\frac{1}{2}\left(B_{h}\circ F_{s}\right)1-\frac{1}{2}\left(B_{h}\circ F_{s}\right)1\right)+v^{T}V_{f}^{T}Bf^{*}\right)\\ \; & =-{\tilde{v}}^{T}\left(S_{h}\circ F_{s}\right)1+1^{T}\left(S_{h}\circ F_{s}\right)\tilde{v}-v^{T}V_{f}^{T}Bf^{*}\\ \; & =-\psi_{L}+\psi_{R}-\left(-{\tilde{v}}_{L}f_{L}^{*}+{\tilde{v}}_{R}f_{R}^{*}\right)\\ \; & =-\left({\tilde{v}}_{R}f_{R}^{*}-\psi_{R}+\psi_{L}-{\tilde{v}}_{L}f_{L}^{*}\right). \end{array}$$

The above equations indicate that the total entropy at the smooth cells is only to the numerical fluxes at the interface of the cell and has no relationship with the fluxes inside the cell. If the numerical fluxes are entropy-stable, then this scheme satisfies the entropy condition.

We sum the total entropy of the $D_{1}$ and $D_{2}$ intervals to obtain the total entropy estimation of the hybrid scheme in the $D_{1}$ and $D_{2}$ intervals.

(34) $$\begin{array}{cc} JW\frac{\partial S}{\partial t} & =JW\frac{\partial S^{SL}}{\partial t}+JM\frac{\partial S^{DG}}{\partial t}\\ \; & =-\left(-v_{0}f_{L,0}^{*}+v_{N}f_{N,R}^{*}-\psi_{N,R}+\psi_{L,0}\right)-\left({\tilde{v}}_{R}f_{R}^{*}-\psi_{R}+\psi_{L}-{\tilde{v}}_{L}f_{L}^{*}\right)\\ \; & =-\left(v_{N}f_{N,R}^{*}-\psi_{N,R}-{\tilde{v}}_{L}f_{L}^{*}+\psi_{L}\right)-\left(-v_{0}f_{L,0}^{*}+\psi_{L,0}+{\tilde{v}}_{R}f_{R}^{*}-\psi_{R}\right). \end{array}$$

where $f_{L,0}^{*}$ and $f_{R}^{*}$ are the interfacial numerical fluxes of the adjacent troubled and smooth cells, which are the same numerical fluxes, and Equation (34) can be simplified as

(35) $$JW\frac{\partial S}{\partial t}=-\left(v_{N}f_{N,R}^{*}-\psi_{N,R}-{\tilde{v}}_{L}f_{L}^{*}+\psi_{L}\right)-\left(\left({\tilde{v}}_{R}-v_{0}\right)f_{R}^{*}-\left(\psi_{R}-\psi_{L,0}\right)\right).$$

The hybrid scheme based on subcell limiting not only requires the scheme to be conservative but also needs to satisfy the entropy-stable condition. Therefore, we must carefully choose the interfacial numerical flux to satisfy the above two conditions simultaneously. If the numerical flux is capable of entropy dissipation at each cell interface, then

(36) $$\left({\tilde{v}}_{0}-v_{R}\right)f^{*ES}-\left(\psi_{L,0}-\psi_{R}\right)\leq 0.$$

Therefore, if the numerical flux is selected to be an entropy-stable numerical flux, the change in the total entropy of the hybrid scheme over time is only related to the interface flux, and the total entropy of the cells gradually decreases with advance calculation, which already indicates that the hybrid scheme satisfies the entropy condition. In conclusion, when appropriate numerical fluxes are selected, the ESDGSEM based on the subcell restriction can satisfy discrete conservation laws and the second law of thermodynamics. Of course, the above theoretical proof is carried out only at the one-dimensional level; the two-dimensional proof process is similar to the one-dimensional proof, which provides the possibility of generalization to two-dimensional spaces.

## 4. Numerical Validation

In this section, we test the de-aliasing properties and shock-capturing capabilities of the schemes through several cases. The isentropic vortex problem is used to test the accuracy of the schemes. The Shu–Osher problem, double Mach reflection problem and transonic flows around the NACA0012 airfoil problem are used to test the shock-capturing capabilities of the ESDGSEM-LG-SL scheme. In addition, the steady shock problem, under-resolved vortical flows and Kelvin–Helmholtz instability problem are used to compare the stability of different high-order schemes.

### 4.1. Shu–Osher

The Shu–Osher problem [39] mainly describes the interaction of sine waves with right-moving shock waves. This case is used to evaluate the ability of the schemes to capture high-frequency waves and shock waves. The initial conditions are as follows

(37) $$\left(\rho ,u,p\right)=\left\{\begin{array}{cc} (3.857143,2.629369,10.33333), & x\leq -4.0\\ (1.0+0.2\sin(50x),0,1.0), & x>-4.0 \end{array}\right..$$

where the computational domain is [−5, 5], and the computing time is *t* = 1.8. The adjustable parameter of the shock indicator is a=0.05, and the reference solution is calculated by the WENO scheme with DOFs=2048.

**Figure 1:**
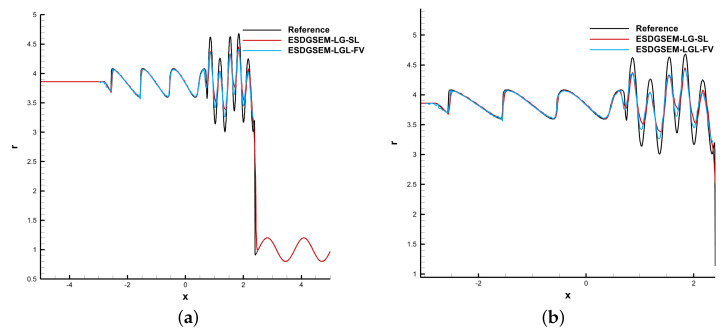
Shu–Osher problem, DOFs=640. (**a**) Global density distribution; (**b**) local density distribution.

The left side of Figure 1 shows the density distributions of the Shu–Osher problem, and the right side shows the local density distributions. ESDGSEM-LGL-FV is the provably entropy-stable subcell shock-capture scheme proposed by Hennemann et al. [30]. Ref. [26] based on the LGL solution points. The ESDGSEM-LG-SL scheme is better in suppressing the numerical oscillations.

### 4.2. Isentropic Vortex

This case is used to test the accuracy of three schemes. In this case, an isentropic vortex disturbance is added to the uniform flow [37]. The uniform flow field is set as

(38) $$\left\{\rho_{\infty },u_{\infty },v_{\infty },p_{\infty }\right\}=\left\{1,1,0,1\right\}$$

The initial conditions of the vortex are set as follows

(39) $$\begin{array}{cc} \Delta u & =-\left(y-y_{c}\right)\frac{\epsilon }{2\pi }\exp\left(\frac{1-r^{2}}{2}\right),\;\Delta v=\left(x-x_{c}\right)\frac{\epsilon }{2\pi }\exp\left(\frac{1-r^{2}}{2}\right),\\ \Delta T & =-\frac{(\gamma -1)\epsilon }{8\gamma \pi^{2}}\exp\left(1-r^{2}\right). \end{array}$$

where the vortex radius is *r*, vortex intensity ϵ=0.5 and vortex centre $\left(x_{c},y_{c}\right)=\left(0.0,0.0\right)$. Through the ideal gas state equation, the initial conditions of the flow field are as follows

(40) $$\left(\rho ,u,v,p\right)=\left[{\left(T_{\infty }+\Delta T\right)}^{\frac{1}{\gamma -1}},u_{\infty }+\Delta u,v_{\infty }+\Delta v,{\left(T_{\infty }+\Delta T\right)}^{\frac{\gamma }{\gamma -1}}\right].$$

where the computational domain is [−10,10]×[−10,10] and the computing time is *t* = 0.1. The polynomial order is 2, and the number of computational grids is set as K×K. The boundary conditions are periodic, and the HLLC flux is used.

Table 1, Table 2 and Table 3 are the error and accuracy orders of the three high-order schemes, respectively. The results show that the high-order schemes can satisfy the expected accuracy, and the ESDGSEM-LG scheme has higher precision.

### 4.3. Steady Shock Wave

This case is mainly used to test the differences of the three schemes in solving the steady shock-wave problem [40]. In this case, the initial conditions are as follows

(41) $$U_{L}=\left(\begin{array}{c} 1\\ 1\\ 0\\ \frac{1}{\gamma \left(\gamma -1\right){\mathrm{Ma}}^{2}}+\frac{1}{2} \end{array}\right),\;U_{R}=\left(\begin{array}{c} f(\mathrm{Ma})\\ 1\\ 0\\ \frac{g(\mathrm{Ma})}{\gamma \left(\gamma -1\right){\mathrm{Ma}}^{2}}+\frac{1}{2f(\mathrm{Ma})} \end{array}\right),$$

where the *L*’ means before the shock wave, and *R* means after the shock wave. Ma represents the Mach number. The f(Ma) and g(Ma) are expressed as

(42) $$\begin{array}{c} f\left(\mathrm{Ma}\right)={\left[\frac{2}{\left(\gamma -1\right){\mathrm{Ma}}^{2}}+\frac{\gamma -1}{\gamma +1}\right]}^{-1},\\ g\left(\mathrm{Ma}\right)=\frac{2\gamma {\mathrm{Ma}}^{2}}{\gamma +1}-\frac{\gamma -1}{\gamma +1}. \end{array}$$

The density, velocity and pressure at the shock location are calculated by the following equations

(43) $$\begin{array}{ccc} \; & \rho_{M}=\rho_{L}+\alpha_{\rho }\left(\rho_{R}-\rho_{L}\right),\; & u_{M}=u_{L}+\alpha_{u}\left(u_{R}-u_{L}\right),\\ \; & v_{M}=v_{L}+\alpha_{v}\left(v_{R}-v_{L}\right),\; & p_{M}=p_{L}+\alpha_{p}\left(p_{R}-p_{L}\right), \end{array}$$

where

(44) $$\begin{array}{cc} \; & \alpha_{\rho }=\epsilon ,\;\alpha_{p}=\epsilon {\left(1+\left(1-\epsilon \right)\frac{\gamma +1}{\gamma -1}\frac{{\mathrm{Ma}}^{2}-1}{{\mathrm{Ma}}^{2}}\right)}^{-\frac{1}{2}},\alpha_{v}=0,\\ \; & \alpha_{u}=1-\left(1-\epsilon \right){\left(1+\epsilon \frac{{\mathrm{Ma}}^{2}-1}{1+\frac{\gamma -1}{2}{\mathrm{Ma}}^{2}}\right)}^{-\frac{1}{2}}{\left(1-\epsilon \frac{{\mathrm{Ma}}^{2}-1}{\frac{2\gamma }{\gamma -1}{\mathrm{Ma}}^{2}-1}\right)}^{-\frac{1}{2}}. \end{array}$$

where ϵ is the shock position, which can be chosen from 0.0 to 1.0. The computational domain is [0,1], and the number of DOFs is 3600. The polynomial order is 2, and the boundary is the Dirichlet boundary. A fixed time step Δt=0.00002 is used, and the computing time is *t* = 50. The adjustable parameter of the shock indicator is a=0.5. The HLL flux is used.

The results are divided into four categories: (i) calculation convergence (oscillation at approximately 1.0× 10${}^{-3}$, where the shock wave position and physical parameters in the calculated results do not fluctuate); (ii) calculation non-convergence (residual oscillation or post-shock fluctuations); (iii) shock wave position not shifted; and (iv) shock wave position shifted (shock wave is shifted from the initial position).

Table 4 and Table 5 show a quality assessment and properties of the numerical solutions, respectively. Table 6 lists the characteristics of the three entropy-stable schemes for the different shock wave positions, in which the Mach number of the fixed shock wave is 2. Table 5 presents the physical characteristics of the three schemes upon increasing the Mach number of the steady shock wave for shock position ϵ=0.5.

As shown in Table 6, both ESDGSEM-LGL and ESDGSEM-LG-SL can be calculated stably and converge, while the ESDGSEM-LG shock positions do not shift and cannot converge. In Table 7, the highest Mach number at which ESDGSEM-LG-FV can be calculated is 10.0, and the physical characteristics meet the expectations. In conclusion, the ESDGSEM-LG-SL scheme based on the subcell restriction performs better regardless of the shock wave intensity or position. Moreover, for the entropy-stable scheme with different point configurations, the scheme at the LGL solution points has better physical characteristics than at the LG solution points in the steady shock problem.

### 4.4. Under-Resolved Vortical Flows

At low Mach numbers, different numerical fluxes have different properties, which may lead to instability in the numerical simulations. Therefore, this example evaluates the performance of the high-order scheme with different numerical fluxes for the under-resolved problem. We use the compressible Euler equations with unsteady inlet boundary conditions mimicking a passive generator (physical screen) of eddies propagating downstream [41]. The domain is initialized with

(45) $$\begin{array}{cc} \; & \rho =\rho_{\infty },\\ \; & \rho u=\rho_{\infty }u_{\infty }\left[1+A\sin\left(ky\right)\sin\left(\Omega t\right)\right],\rho v=0,\\ \; & E=p_{\infty }/\left(\gamma -1\right)+\rho_{\infty }u_{\infty }^{2}/2. \end{array}$$

where $\rho_{\infty }=1,u_{\infty }=1$ is the free flow density and velocity, and $p_{\infty }=\rho_{\infty }c_{\infty }^{2}/\gamma$ is the free flow pressure. In addition, disturbance parameters A=1/2,K=5 and Ω=1 are defined for the initial condition. The outflow boundary is consistent with the initial conditions, and the upper and lower boundaries are inviscid wall boundaries. The grid number is 120×12, and the computational domain is Ω=[0,20π]×[−π,π]. The polynomial order is 3. The CFL is 0.5, and the computing time is *t* = 150. The numerical solution of the flow field is stable when *t* = 20 π.

In this case, different Riemann solvers have different characteristics for high-order schemes, resulting in different performances for solving the under-resolved vortical flow problem. We mainly compare two aspects: (i) the time the simulation remains stable and (ii) vorticity structures in the flow field, calculated by $w=\frac{\partial v}{\partial x}-\frac{\partial u}{\partial y}$.

Table 8 shows the stable computation time of the four high-order schemes using different numerical fluxes. The high-order schemes without the entropy-stable strategy undergo computation collapse under the LLF numerical flux, while the other schemes acquire stable calculations. Therefore, the entropy-stable strategy is more stable for low Mach number under-resolved flows.

**Figure 2:**
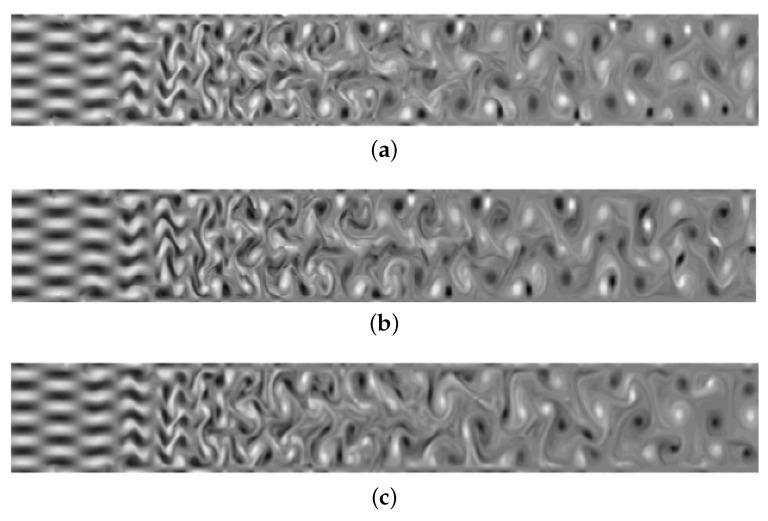
Using the HLL flux vorticity field. (**a**) DGSEM-LGL; (**b**) ESDGSEM-LGL; (**c**) ESDGSEM-LG.

**Figure 3:**
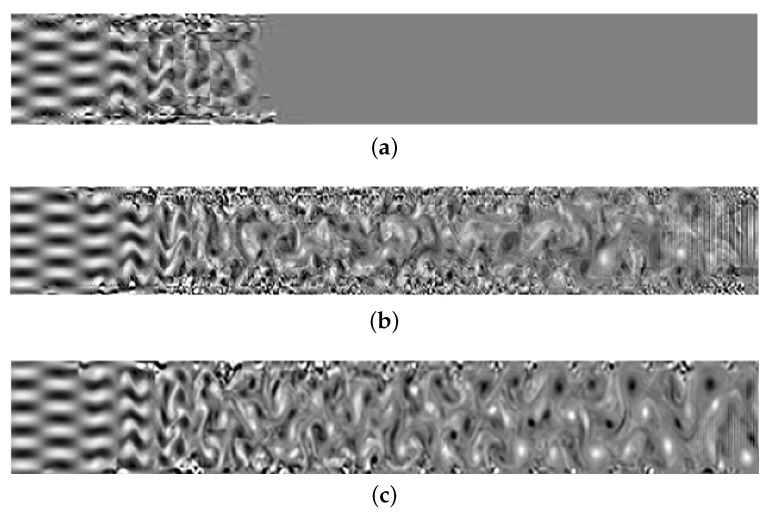
Using the LLF flux vorticity field. (**a**) DGSEM-LGL; (**b**) ESDGSEM-LGL; (**c**) ESDGSEM-LG.

Figure 2 and Figure 3 compare the stability and resolution of several high-order schemes as evaluated by the vorticity of the flow field. Figure 3a shows the vorticity distribution before the collapse of the DGSEM-LGL scheme when *t* = 23.6, and Figure 3b,c show the vorticity distributions are calculated statically with high-entropy stability under different node configurations. The LLF numerical flux produces spurious reflections near the outflow boundary, and the entropy-stable strategy has an effect in suppressing the instability. Moreover, Figure 3b,c also show that the entropy-stable strategy based on the LG solution points has good robustness and resolution.

### 4.5. Kelvin–Helmholtz Instability

To test the stability of the high-order schemes, we simulated the Kelvin–Helmholtz instability problem [38]. This case is very challenging to calculate flow fields for high-order schemes, such as the DG scheme. The initial conditions are as follows

(46) $$\begin{array}{cc} \rho =\frac{1}{2}+\frac{3}{4}B, & p=1,\\ u=\frac{1}{2}\left(B-1\right), & v=\frac{1}{10}\sin\left(2\pi x\right), \end{array}$$

where B(x,y) is a smooth approximation to a discontinuous function,

(47) B(x,y)=tanh(15y+7.5)−tanh(15y−7.5).

where the computation domain is [−1,1]×[−1,1], and the period boundary condition is used. The adjustable parameter of the shock indicator is a=0.5, and the CFL number is 0.5. The grid size is 120×12. The polynomial order is 3. Furthermore, to show the evolution of the total entropy over time in the whole numerical simulation, we calculate the total entropy as

$$S\left(t\right)=-\int_{\Omega }\frac{\rho S}{\lambda -1},$$

where $s=\log\left(\frac{p}{\rho^{\lambda }}\right)$ and the minus sign means that if the high-order scheme satisfies the second law of thermodynamics, its total entropy decreases gradually throughout the numerical simulation [42].

**Figure 4:**
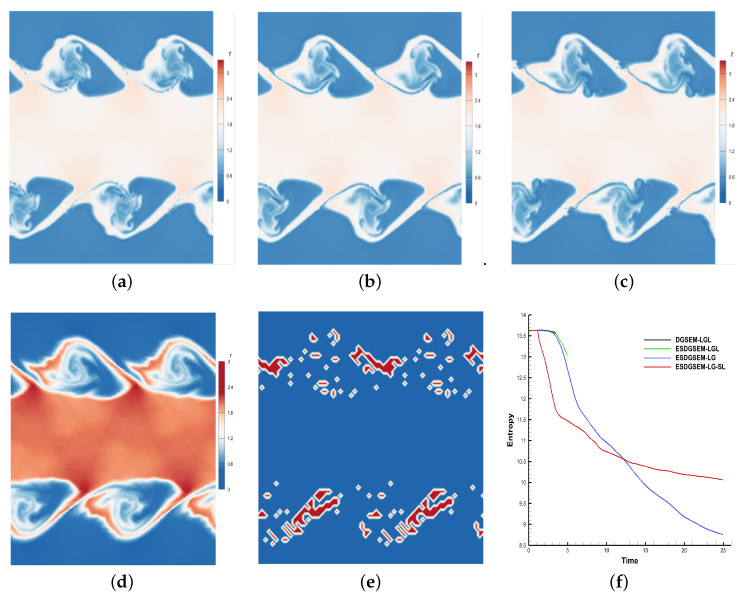
Density distribution and total entropy evolution. (**a**) DGSEM-LGL; (**b**) ESDGSEM-LGL; (**c**) ESDGSEM-LG; (**d**) ESDGSEM-LG-SL; (**e**) troubled cell distribution; (**f**) total entropy evolution.

Figure 4 shows the different performances of the four high-order schemes for calculating instability problems, where the computational time of Figure 4a–d is 3.7 and the computational termination time of Figure 4f is 25. As seen from the comparison of Figure 4a–c, the high-order scheme using the entropy-stable strategy is more robust than the traditional high-order scheme, and the high-order entropy-stabilization strategy based on subcell limiting performs better in suppressing oscillations. Figure 4f mainly describes the evolution process of the total entropy of the four schemes till *t* = 25.0. Among the schemes, the DGSEM-LGL and ESDGSEM-LGL schemes exhibit computational collapse due to the absence of the stability strategy, whereas the other schemes satisfy the second law of thermodynamics.

Table 9 mainly shows the physical characteristics of the high-Reynolds-number instability problem calculated by the four high-order schemes when the calculation time is extended and different numerical fluxes are adopted.

The following conclusions can be drawn from the example of two-dimensional Kelvin–Helmholtz instability problems: (i) the entropy-stability strategy is more robust in calculating instability problems; (ii) for the Kelvin–Helmholtz instability problem, the entropy-stability strategy based on LG solution points has a higher resolution than that of LGL solution points; (iii) the hybrid scheme can satisfy the condition of entropy stability both theoretically and numerically; (iv) the hybrid scheme has a greater advantage in suppressing oscillations.

### 4.6. Double Mach Reflection

The double Mach reflection problem is a popular test case to evaluate the strong shock-capturing capability of high-order schemes [43]. In this section, the double Mach example is used to test the shock-capture capability of high-order entropy-stable schemes based on subcell limiting. The computational domain is [0, 4] × [0, 1]. The grid number is 240×60, and the polynomial order is 3. The adjustable parameter of the shock indicator is a=0.05, and the HLL flux is selected. The simulation starts at the bottom boundary of the domain at x = 1/6, with an angle of 60 degrees to the boundary. A shock wave with a Mach number of 10 moves from left to right. The computing time is *t* = 0.2, during which two Mach stems and a strong shock wave is generated.

**Figure 5:**
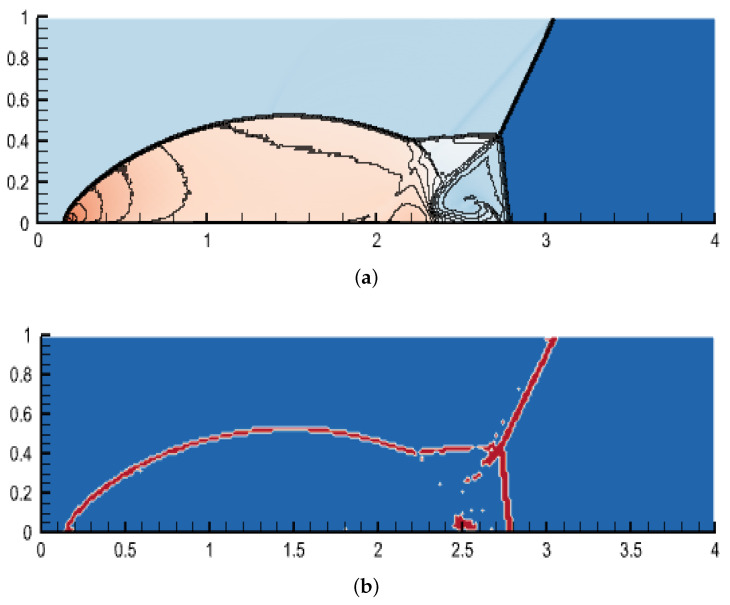
Density contour and troubled cell distribution for the double Mach reflection problem. (**a**) Density; (**b**) troubled cell distribution.

A density contour and troubled cell distribution of the double Mach problem show that the basic structure of the flow field is captured, as shown in Figure 5. This shows that the high-order entropy-stable scheme based on subcell limiting can describe the subtle changes in the flow field, although there are some numerical oscillations.

### 4.7. Transonic Flows around the NACA0012 Airfoil

The purpose of this example is to simulate the transonic flow around the NACA0012 airfoil [44] and test the shock-capture ability and convergence performance of the high-order entropy-stabilized DG method for the curved boundary problem. The angle of attack is 1.25 degrees. For transonic inflow with Mach 0.8, the numbers of grid cells distributed in the circumferential and radial directions are 120 and 80, respectively. The polynomial order is 2, and the computational time *t* is 50 with time steps determined by CFL = 0.5. The adjustable parameter of the shock indicator is a=0.001. The LLF flux is used.

**Figure 6:**
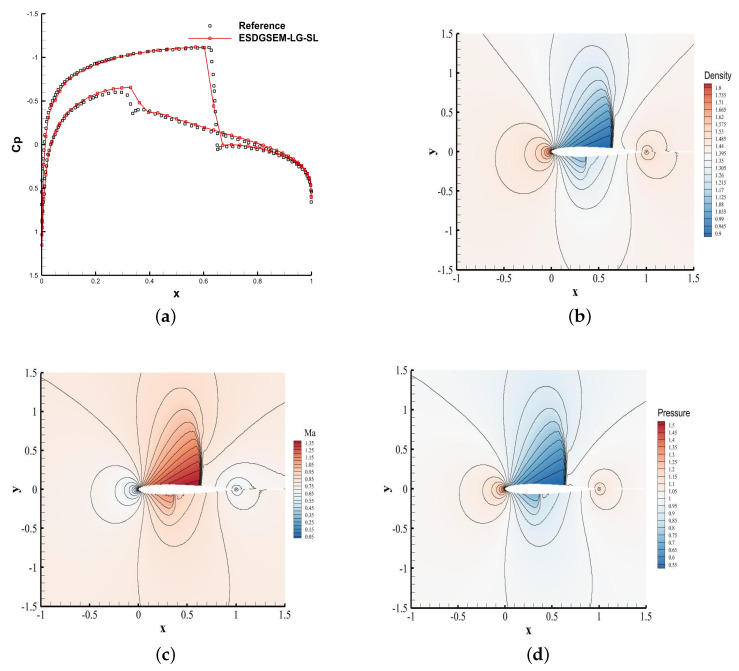

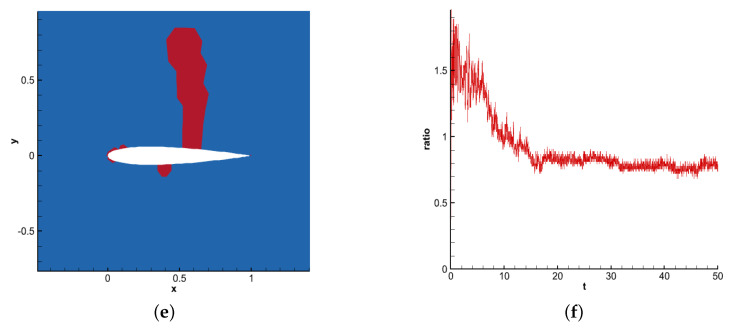
The results of the NACA0012 airfoil. (**a**) Pressure coefficient; (**b**) density; (**c**) Mach number; (**d**) pressure; (**e**) troubled cell distribution; (**f**) ratio of troubled cells.

As shown in Figure 6, the ESDGSEM-LG-SL scheme is used to simulate the airfoil problem, and the density, pressure, Mach number, surface pressure coefficient and troubled cell distribution are obtained. Figure 6e,f show that the scheme correctly identifies the troubled cells. The contour results of Figure 6b–d show that the shock waves on the upper and lower surfaces of the wing are well captured. As shown in Figure 6a, the pressure coefficients of the upper and lower surfaces exhibit less numerical oscillation near the discontinuity point. Therefore, the scheme can predict the apparent pressure coefficient of the airfoil accurately.

## 5. Conclusions

In this work, a shock-capturing strategy for the DGSEM method based on subcell limiting provable entropy stability is proposed at LG solution points. The main idea of this paper is to, firstly, divide the solution regions into smooth and troubled regions by a shock indicator; then, use a high-order scheme for smoothed regions and a low-order scheme for troubled regions. To ensure that the hybrid scheme satisfies the entropy conservative condition or the entropy-stable condition, it is necessary to select the same entropy conservative flux or entropy-stable flux.

Through numerical tests, we obtain the following conclusions:

1. The DGSEM methods based on the entropy-stable strategy have better robustness when solving under-resolved flows than the traditional DGSEM method.
2. The ESDGSEM-LG-SL scheme can stably calculate high-Mach-number problems, indicating that the scheme has good stability. Furthermore, the results of the double Mach reflection problem and transonic flows around the NACA0012 airfoil case also show that this scheme has a better shock-capturing ability than the other schemes.

At present, the detailed theoretical proof of this theory is only given for the structural mesh; further work is needed for the unstructured mesh. Future planned work focuses on how to extend the provable entropy-stable subcell limiting technique to reach second-order precision.

## Figures and Tables

**Table 1:** The accuracy test of the DGSEM scheme.

K×K	$L_{1}$ Error	Order	$L_{2}$ Error	Order	$L_{\infty }$ Error	Order
10×10	9.0244× 10${}^{-4}$		3.6951× 10${}^{-3}$		2.8658× 10${}^{-2}$	
20×20	2.1134× 10${}^{-4}$	2.99	9.6161× 10${}^{-4}$	2.98	1.1164× 10${}^{-2}$	2.92
40×40	5.1408× 10${}^{-5}$	3.02	2.4907× 10${}^{-4}$	3.00	4.4411× 10${}^{-3}$	2.98
80×80	9.4452× 10${}^{-6}$	3.01	4.7200× 10${}^{-5}$	3.00	9.4786× 10${}^{-4}$	3.00

**Table 2:** The accuracy test of the ESDGSEM-LGL scheme.

K×K	$L_{1}$ Error	Order	$L_{2}$ Error	Order	$L_{\infty }$ Error	Order
10×10	8.7745× 10${}^{-4}$		3.5893× 10${}^{-3}$		2.7553× 10${}^{-2}$	
20×20	2.0834× 10${}^{-4}$	2.99	9.3536× 10${}^{-4}$	2.98	1.3921× 10${}^{-2}$	2.92
40×40	4.9329× 10${}^{-5}$	3.02	2.3544× 10${}^{-4}$	3.00	3.9784× 10${}^{-3}$	2.98
80×80	8.7955× 10${}^{-6}$	3.01	4.3345× 10${}^{-5}$	3.00	9.0972× 10${}^{-4}$	3.00

**Table 3:** The accuracy test of the ESDGSEM-LG scheme.

K×K	$L_{1}$ Error	Order	$L_{2}$ Error	Order	$L_{\infty }$ Error	Order
10×10	4.3711× 10${}^{-4}$		1.8270× 10${}^{-3}$		1.7069× 10${}^{-2}$	
20×20	7.4065× 10${}^{-5}$	3.02	3.4896× 10${}^{-4}$	3.02	5.1147× 10${}^{-3}$	2.98
40×40	1.2349× 10${}^{-5}$	3.01	5.5574× 10${}^{-5}$	3.00	1.0048× 10${}^{-3}$	2.99
80×80	1.7967× 10${}^{-6}$	3.00	8.5565× 10${}^{-6}$	3.00	1.6041× 10${}^{-4}$	3.00

**Table 4:** Quality assessment of the numerical solutions.

Case	(0, 0)	(0, 1)	(1, 0)	(1, 1)
Number	0	1	2	3

**Table 5:** Properties of the numerical solutions.

Computational Convergence	Position of the Shock Wave Is Not Shifted
Yes: 1	Yes: 1
No: 0	No: 0

**Table 6:** The influence of the shock position (Ma = 2.0).

Schemes	0.0	0.2	0.4	0.6	0.8	1.0
ESDGSEM-LGL	3	3	3	3	3	3
ESDGSEM-LG	1	1	1	1	1	1
ESDGSEM-LG-SL	3	3	3	3	3	3

**Table 7:** The influence of the Mach number (ϵ=0.5).

Schemes	1.0	2.0	4.0	6.0	8.0	10.0
ESDGSEM-LGL	3	3	3	3	0	0
ESDGSEM-LG	3	1	1	1	1	1
ESDGSEM-LG-SL	3	3	3	3	3	3

**Table 8:** Stable computation time.

Schemes	LLF	HLL
DGSEM-LGL	23.6	150
ESDGSEM-LGL	150	150
ESDGSEM-LG	150	150
ESDGSEM-LG-SL	150	150

**Table 9:** Stable computation time.

Schemes	LLF	HLL
DGSEM-LGL	3.16	4.72
ESDGSEM-LGL	6.26	5.02
ESDGSEM-LG	25.0	25.0
ESDGSEM-LG-SL	25.0	25.0

## Data Availability

Not applicable.

## Appendix A

The key to construct an entropy-stable scheme is to select an appropriate convex entropy. For two-dimensional Euler equations, the entropy function has been given. The formula of the entropy variable [21] is as follows

(A1) $$\begin{array}{cc} \; & v_{1}=\frac{\rho e(\lambda +1-s)-E}{\rho e},\\ \; & v_{1+i}=\frac{\rho u_{i}}{\rho e},\;i=1,2,\\ \; & v_{4}=-\frac{\rho }{\rho e}, \end{array}$$

We can derive the entropy variable in terms of the conserved variable as

(A2) $$\begin{array}{cc} \; & \rho =-\left(\rho e\right)v_{4},\\ \; & \rho u_{i}=\left(\rho e\right)v_{1+i},\;i=1,2,\\ \; & E=\left(\rho e\right)\left(1-\frac{\sum_{j=1}^{2}v_{1+j}^{2}}{2v_{4}}\right), \end{array}$$

where $\rho e={\left(\frac{\left(\lambda -1\right)}{{\left(-v_{4}\right)}^{\lambda }}\right)}^{\frac{1}{\left(\lambda -1\right)}}e^{\frac{-s}{\lambda -1}},\;s=\lambda -v_{1}+\frac{\sum_{j=1}^{2}v_{1+j}^{2}}{2v_{4}}$.

Therefore, the two-dimensional entropy conservative flux is expressed as

(A3) $$\begin{array}{cc} f_{1,s}^{1}\left(u_{L},u_{R}\right)={\left\{\left\{\rho \right\}\right\}}^{\log}\left\{\left\{u_{1}\right\}\right\}, & f_{2,s}^{1}\left(u_{L},u_{R}\right)={\left\{\left\{\rho \right\}\right\}}^{\log}\left\{\left\{u_{2}\right\}\right\},\\ f_{1,s}^{2}\left(u_{L},u_{R}\right)=f_{1,s}^{1}\left\{\left\{u_{1}\right\}\right\}+p_{\mathrm{avg}\;}, & f_{2,s}^{2}\left(u_{L},u_{R}\right)=f_{2,s}^{1}\left\{\left\{u_{1}\right\}\right\},\\ f_{1,s}^{3}\left(u_{L},u_{R}\right)=f_{2,s}^{2}, & f_{3,s}^{3}\left(u_{L},u_{R}\right)=f_{2,s}^{1}\left\{\left\{u_{2}\right\}\right\}+p_{\mathrm{avg}\;},\\ f_{1,s}^{4}\left(u_{L},u_{R}\right)=\left(E_{\mathrm{avg}\;}+p_{\mathrm{avg}\;}\right)\left\{\left\{u_{1}\right\}\right\}, & f_{2,s}^{4}\left(u_{L},u_{R}\right)=\left(E_{\mathrm{avg}\;}+p_{\mathrm{avg}\;}\right)\left\{\left\{u_{2}\right\}\right\}, \end{array}$$

with auxiliary parameters

(A4) $$\begin{array}{cc} \; & \beta =\frac{\rho }{2p},\;p_{\mathrm{avg}\;}=\frac{\left\{\{\rho \}\right\}}{2\{\{\beta \}\}},\;E_{\mathrm{avg}\;}=\frac{{\left\{\left\{\rho \right\}\right\}}^{\log}}{2\left(\lambda -1\right){\left\{\left\{\beta \right\}\right\}}^{\log}}+\frac{1}{2}{\left\{\left\{\rho \right\}\right\}}^{\log}u_{\mathrm{avg}\;}^{2},\\ \; & u_{\mathrm{avg}\;}^{2}=2\left({\left\{\left\{u_{1}\right\}\right\}}^{2}+{\left\{\left\{u_{2}\right\}\right\}}^{2}\right)-\left(\left\{\left\{u_{1}^{2}\right\}\right\}+\left\{\left\{u_{2}^{2}\right\}\right\}\right). \end{array}$$

To calculate the entropy conservative flux, it is necessary to calculate the mean value of the parameters and the logarithmic mean value. Let *f* represent the piecewise polynomial function, and $f^{+}$ represent the corresponding function value of *f* outside the cell interface. In addition, we can define the mean value and logarithmic mean value of the function as [45]

(A5) $$\left\{\left\{f\right\}\right\}=\frac{f^{+}+f}{2},\;{\left\{\left\{f\right\}\right\}}^{\log}=\frac{f^{+}-f}{\log\left(f^{+}\right)-\log\left(f\right)}.$$
